# Multiplex giant magnetoresistive biosensor microarrays identify interferon-associated autoantibodies in systemic lupus erythematosus

**DOI:** 10.1038/srep27623

**Published:** 2016-06-09

**Authors:** Jung-Rok Lee, D. James Haddon, Hannah E. Wand, Jordan V. Price, Vivian K. Diep, Drew A. Hall, Michelle Petri, Emily C. Baechler, Imelda M. Balboni, Paul J. Utz, Shan X. Wang

**Affiliations:** 1 Department of Mechanical Engineering, Stanford University, Stanford, California, USA; 2 Department of Medicine, Division of Immunology and Rheumatology, Stanford University, Stanford, California, USA; 3 Department of Molecular and Cell Biology, Division of Immunology and Pathogenesis, University of California, Berkeley, California, USA; 4 Department of Electrical and Computer Engineering, University of California, San Diego, California, USA; 5 Division of Rheumatology, Johns Hopkins University School of Medicine, Baltimore, Maryland, USA; 6 Center for Immunology, University of Minnesota Medical School, Minneapolis, Minnesota, USA; 7 Department of Pediatrics, Division of Allergy, Immunology and Rheumatology, Stanford University, Stanford, California, USA; 8 Institute for Immunity, Transplantation and Infection, Stanford University School of Medicine, Stanford, California, USA; 9 Department of Materials Science and Engineering, Stanford University, Stanford, California, USA; 10 Department of Electrical Engineering, Stanford School of Engineering, Stanford, California, USA

## Abstract

High titer, class-switched autoantibodies are a hallmark of systemic lupus erythematosus (SLE). Dysregulation of the interferon (IFN) pathway is observed in individuals with active SLE, although the association of specific autoantibodies with chemokine score, a combined measurement of three IFN-regulated chemokines, is not known. To identify autoantibodies associated with chemokine score, we developed giant magnetoresistive (GMR) biosensor microarrays, which allow the parallel measurement of multiple serum antibodies to autoantigens and peptides. We used the microarrays to analyze serum samples from SLE patients and found individuals with high chemokine scores had significantly greater reactivity to 13 autoantigens than individuals with low chemokine scores. Our findings demonstrate that multiple autoantibodies, including antibodies to U1-70K and modified histone H2B tails, are associated with IFN dysregulation in SLE. Further, they show the microarrays are capable of identifying autoantibodies associated with relevant clinical manifestations of SLE, with potential for use as biomarkers in clinical practice.

Systemic lupus erythematous (SLE) is a chronic, inflammatory autoimmune disease that affects multiple organ systems, with an estimated prevalence of 300,000 people in the United States^1^. The manifestations of SLE are heterogeneous, making it a difficult disease to manage clinically. Further, SLE has an unpredictable course, with periods of flares and remission. A hallmark of SLE is the presence of high titer, class-switched antibodies that bind nuclear antigens, including DNA, ribonucleoprotein (RNP), Smith, Ro, La, and histones^2^,^3^. Gene expression microarray analysis of the peripheral blood mononuclear cells (PBMCs) of individuals with SLE has shown that the interferon (IFN) pathway is dysregulated in a subset of individuals who have more severe disease^4^. Subsequently, the chemokine score, based on serum levels of three interferon-regulated chemokines, was found to be positively correlated with the interferon signature, disease activity, and likelihood of flare^5^,^6^. Identification of autoantibodies that are associated with elevated chemokine scores could increase our understanding of the mechanisms leading to dysregulation of the IFN pathway in SLE, and the causes of disease flares.

At least 180 autoantigens have been described in SLE^7^. However, current clinical tests typically only measure levels of a single autoantibody, potentially missing much of the clinical heterogeneity of SLE. Autoantigen microarrays have the advantage of profiling hundreds of autoantibodies in parallel, but typically take multiple days to perform^8^. Giant magnetoresistive (GMR) biosensors are microscopic electrical sensors that can detect local magnetic field changes induced by the presence of magnetic nanoparticles (MNPs) in their proximity^9^,^10^,^11^,^12^. Recently, our group developed multiplexed assays for protein biomarkers by coating GMR biosensors with capture antibodies and MNPs with detection antibodies^9^,^13^,^14^. The GMR biosensors were highly sensitive, had a large dynamic range (>4 decades)^9^, and allowed for the real-time measurement of antibody binding^15^. Real-time measurement has the added benefit of making GMR biosensor assays faster to perform than traditional fluorescence-based microarrays. To date, GMR biosensors have not been applied to the multiplexed detection of autoantibodies.

In this study, we developed GMR biosensor microarrays for the multiplexed measurement of antibodies to known autoantigens, including post-translationally modified (PTM) peptides. We used the GMR biosensor autoantigen microarrays to identify autoantibodies that were significantly associated with elevated chemokine scores in individuals with SLE. Elucidating the relationship between autoantibodies and dysregulation of the IFN pathway may provide new insights into SLE pathogenesis, and enable rapid monitoring of disease activity.

## Results

### Development and validation of GMR biosensor autoantigen microarrays

**Figure 1:**
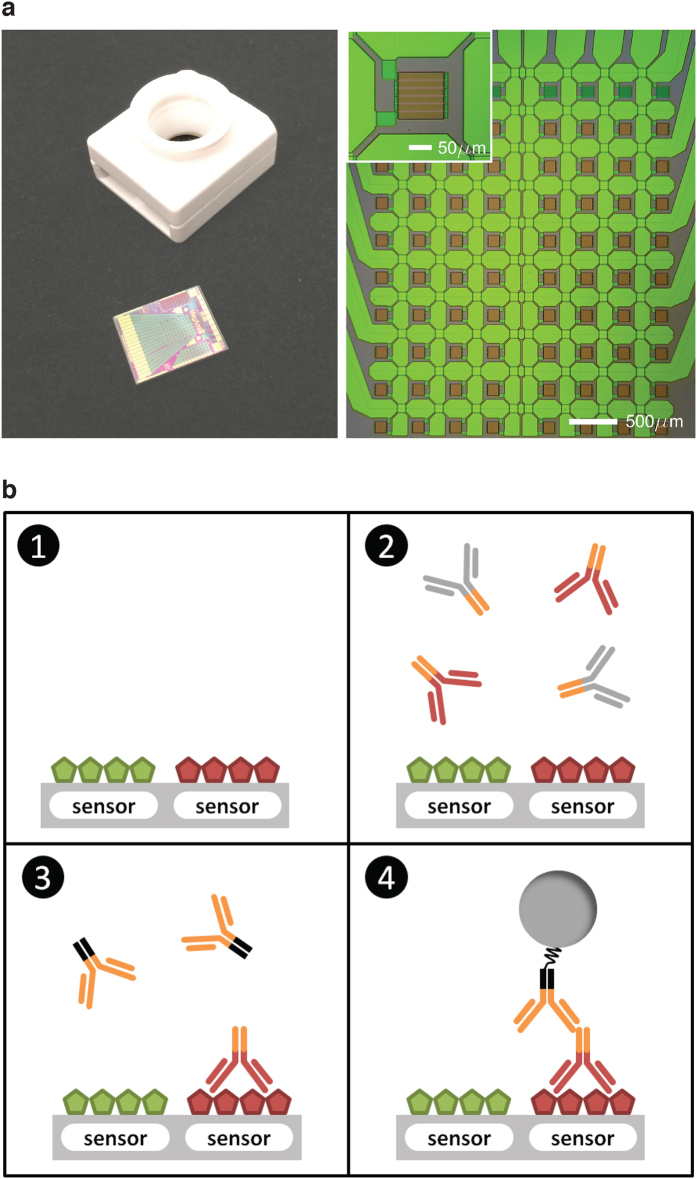
GMR biosensor autoantigen microarrays. (**a**) Optical images of a GMR biosensor chip and a cartridge with a reaction well (left). The sensor chip measures 10 × 12 mm and consists of an array of 8 × 10 sensors (total 80 sensors). Each sensor size is 100 × 100 μm (right). (**b**) A schematic of assaying antibody reactivity to autoantigens (not to scale). (1) Autoantigens were printed on the surface of the chip’s sensors. (2) The sample was added to the reaction well, allowing antibodies to bind to their corresponding antigens. (3) After washing, species-specific, biotinylated anti-IgG antibodies were used as a secondary reagent. (4) Streptavidin-coated MNPs bind to the biotinylated detection antibodies, and the respective sensor detects stray field from the bound MNPs.

To investigate the application of GMR biosensor technology to the multiplexed measurement of autoantibodies, we designed and fabricated GMR biosensor microarray chips, each with 72 effective sensors, as the platform for autoantigen microarrays (Fig. 1a). We used a non-contact robotic microarrayer to print known SLE autoantigens (histones H2A and H4, H2B, and H3, Ribo P, dsDNA, U1-70K, Ro52, Ro60, La/SSB, and Smith) on the surface of the chips’ GMR biosensors. We also printed known antigenic peptides derived from the histone H2B N-terminal tail and the RNA-binding domain of U1-70K (as well as FLAG peptide controls). More detailed information about the selected autoantigens and peptides is presented in Supplementary Tables 1 and 2. A schematic of the analysis of antibody-containing samples using GMR biosensor autoantigen microarrays is shown in Fig. 1b.

**Figure 2:**
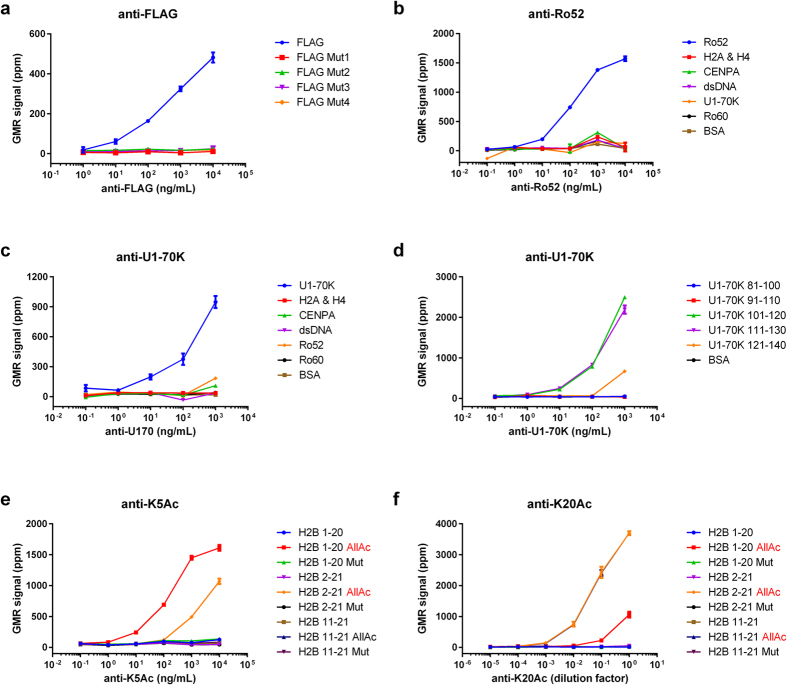
Validation of GMR biosensor autoantigen microarrays with commercial antibodies, including PTM-specific antibodies. Serial dilutions of monoclonal antibodies to (**a**) FLAG, (**b**) Ro52, (**e**) H2B K5Ac, and (**f**) H2B K20Ac, as well as polyclonal antibodies to (**c,d**) U1-70K, were used to probe individual GMR biosensor autoantigen microarrays printed with the cognate antigens and controls. The target epitopes K5Ac and K20Ac, are indicated in red in (**e**,**f**) respectively. Each data point is the average of signals from four replicate sensors on the microarray, and error bars represent standard deviations. The concentration of anti-H2B K20Ac was not provided by the manufacturer, and is presented as a relative concentration to the stock solution.

To validate the GMR biosensor autoantigen microarrays, we started by performing a titration of a monoclonal antibody to the FLAG epitope (M2 clone). We found that anti-FLAG was highly specific to the FLAG octapeptide, and did not bind to any of the FLAG point mutants immobilized on the biosensors of the microarrays (Fig. 2a). The GMR microarrays had dynamic ranges and detection limits that were comparable to peptide ELISA performed with the same antibody and FLAG peptides (Supplementary Figure 1a). Next, we tested the microarrays using monoclonal and polyclonal antibodies to Ro52 and U1-70K, respectively. We found that both antibodies bound to their target antigens, but not to other proteins or dsDNA immobilized on the surface of the microarray (Fig. 2b,c). We also found that the polyclonal U1-70K antibody was reactive to sequences within the RNA-binding domain of U1-70K (Fig. 2d), which were previously reported by our group^16^. These results demonstrate that the GMR biosensor autoantigen microarrays are capable of sensitive and specific detection of the reactivity of monoclonal and polyclonal antibodies to protein and peptide antigens. In addition, we found that the intrinsic charge of MNPs does not influence detection of serum antibodies to charged antigens compared to ELISA (Supplementary Figure 2).

To investigate whether GMR biosensor autoantigen microarrays could be used to measure antibodies to post-translationally modified autoantigens, we probed the microarrays with monoclonal antibodies specific to acetylated forms (K5Ac and K20Ac) of the histone H2B N-terminal tail. The GMR biosensor autoantigen microarrays showed high levels of binding of anti-K5Ac to the two K5Ac modified H2B peptides (H2B 1-20 AllAc and H2B 2-21 AllAc), but not to unmodified and mutated H2B peptides (Fig. 2e). Similarly, anti-K20Ac showed high levels of specific reactivity to the three K20Ac-modified H2B peptides (H2B 1-20 AllAc, H2B 2-21 AllAc and H2B 11-21 AllAc), but not unmodified and mutated H2B peptides (Fig. 2f). Removal of the proline at the N-terminus or the alanine at the C-terminus reduced binding of anti-K5Ac and anti-H2B K20Ac, respectively (Fig. 2e,f). We performed ELISA with the same antibodies and H2B peptides for comparison, and found ELISA had a higher level of non-specific binding to unmodified peptides than was observed with the GMR microarrays (Supplementary Figure 3). These results demonstrate that the GMR biosensor autoantigen microarrays can be used to measure PTM-specific antibodies, and define the critical residues in their consensus binding sequences.

### GMR biosensor autoantigen microarrays identify autoantibodies associated with dysregulation of the IFN pathway in SLE

Dysregulation of the IFN pathway is thought to play a central role in SLE. Chemokine scores, based on the serum levels of three IFN-regulated chemokines, are associated with SLE, disease activity, and likelihood of flare^5^. Knowledge of whether specific autoantibodies are associated with chemokine scores could help clarify the mechanisms leading to dysregulation of the IFN pathway and disease flares in SLE. To identify autoantibodies associated with dysregulation of the IFN pathway, we used GMR biosensor autoantigen microarrays to compare serum samples from 15 SLE patients who had high chemokine scores with 15 SLE patients who had low chemokine scores (Table 1). Chemokine scores were based on serum levels of three IFN-regulated chemokines: MCP1 (Monocyte chemotactic protein 1, CCL2), IP10 (Interferon gamma-induced protein 10, CXCL10), and MIP3β (Macrophage inflammatory protein-3-beta, CCL19)^6^. Ten healthy samples were measured in parallel as controls. For the analysis of patient serum, we modified our method for printing peptide antigens to a two-step process, where we printed streptavidin on each biosensor prior to printing biotinylated peptides (Supplementary Figure 4). We found results from this approach had a higher level of agreement with peptide ELISA, compared to direct spotting of peptides (Supplementary Figure 5). To avoid the possibility of anti-human IgG biotinylated antibodies directly binding to the streptavidin on the surface of the microarrays, we used Protein G-coated MNPs to detect antibody-peptide binding instead^17^. Overall, the GMR biosensor autoantigen microarrays had a high level of agreement with traditional fluorescence-based autoantigen microarrays and clinical tests (Supplementary Figure 6).

**Figure 3:**
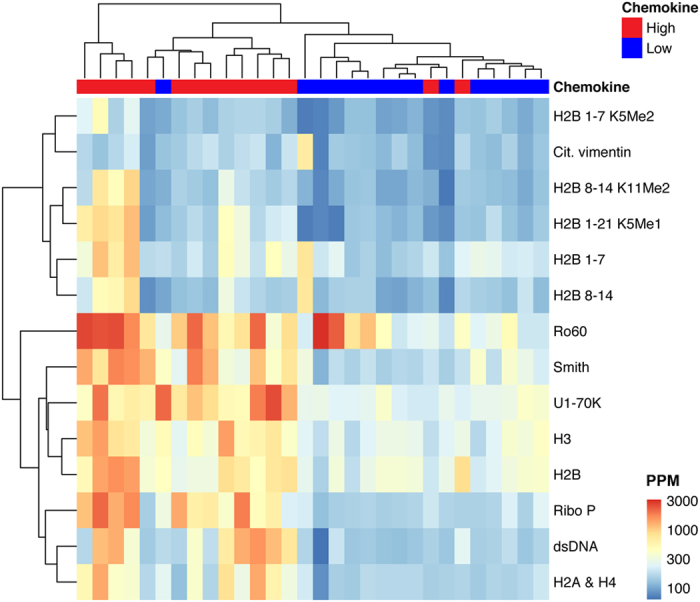
GMR biosensor autoantigen microarrays identify autoantibodies associated with dysregulation of the IFN pathway in SLE. Serum samples from SLE patients with high (n = 15) or low (n = 15) chemokine scores were evaluated using GMR biosensor autoantigen microarrays. A single microarray was used to measure each sample. Significance analysis of microarrays (SAM) was used to identify autoantigens with significantly different antibody reactivity between the groups (q < 0.001, Wilcoxon). An unsupervised hierarchically clustered heatmap (Euclidean distance, complete linkage) shows the candidate autoantigens identified by SAM.

The Significance Analysis of Microarrays (SAM)^18^ algorithm was used to identify statistically significant differences in autoantigen reactivity between the high and low chemokine score groups. SAM accounts for the large number of comparisons inherent in microarray analyses by using repeated random permutations of the data to estimate the false discovery rate (FDR). SAM identified 14 autoantigens with significantly greater reactivity in SLE patients with high chemokine scores than low chemokine scores (Fig. 3), including dsDNA, which had previously been associated with high chemokine scores by Bauer *et al*.^6^. A citrullinated vimentin peptide was found to be significant by SAM, but reactivity levels to the peptide were very low and the most reactive sample was from an individual in the low chemokine score group, so we focused on the other autoantigens. To our knowledge, the other 12 autoantibodies identified by SAM have not previously been associated with chemokine scores. They included histones (H2A and H4, H2B, and H3), methylated (K5Me1, K5Me2 and K11Me2) and unmodified peptides from the N-terminal tail of H2B, Ribo P, Sm, Ro60, and U1-70K.

### Validation of autoantibody associations with dysregulation of the IFN pathway by ELISA

**Figure 4:**
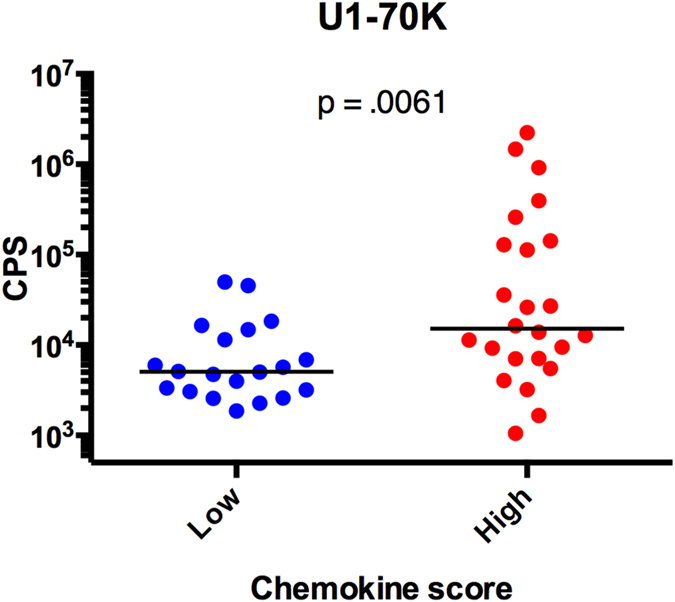
Antibodies to U1-70K are associated with high chemokine scores in SLE. Serum samples from SLE patients with high (n = 24; red) and low (n = 20; blue) chemokine scores were assessed for IgG reactivity to U1-70K by indirect ELISA. P-value was determined using a Mann-Whitney test. Bars represent medians.

To confirm the association of anti-U1-70K with chemokine scores observed by the microarray, we performed ELISA with sera from an independent set of SLE patients in the ABCoN cohort with high (n = 24) and low (n = 20) chemokine scores. In agreement with the GMR biosensor autoantigen microarray, we found that individuals with high chemokine scores had higher levels of serum IgG antibodies to U1-70K, than those with low chemokine scores (Fig. 4).

**Figure 5:**
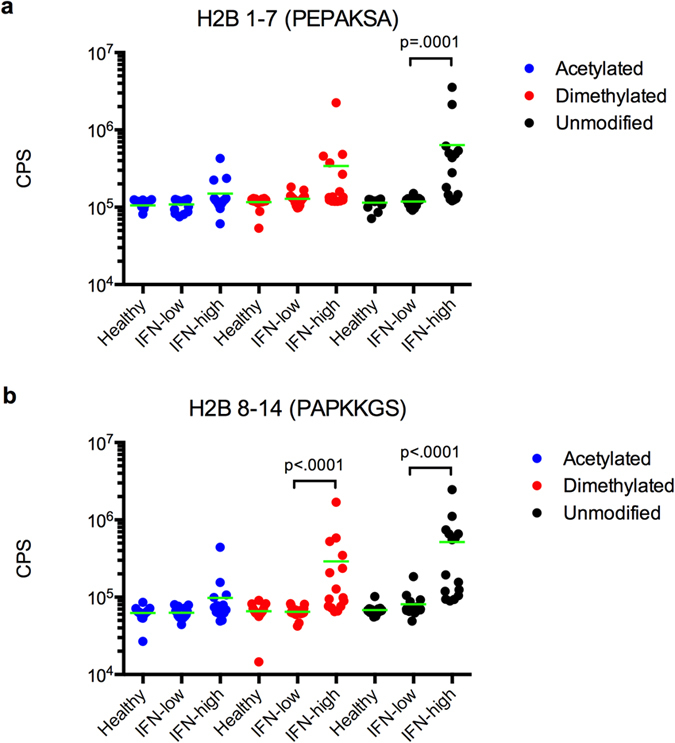
Antibodies to post-translationally modified forms of the H2B N-terminal tail are associated with dysregulation of the IFN pathway. Sera from SLE patients with high (n = 15) and low (n = 15) IFN signatures as well as healthy controls (n = 15) were analyzed for IgG reactivity to peptides from the N-terminal tail of histone H2B by indirect ELISA. The peptides corresponded to acetylated (blue), dimethylated (red) and unmodified (black) forms of H2B 1-7 (**a**) and H2B 8-14 (**b**). P-values were determined using Mann-Whitney tests. Bars represent medians.

To confirm the association of antibodies to methylated and unmodified peptides from the N-terminal tail of histone H2B with dysregulation of the IFN pathway, we performed peptide ELISA with an independent cohort of SLE patients with high (n = 15) and low (n = 15) IFN signatures. The sera of 15 healthy controls were measured in parallel. We selected acetylated, dimethylated and unmodified versions of H2B 1-7 and H2B 8-14 for the ELISA. Similar to observations with the microarrays (Fig. 3 and Supplementary Figure 7), we found that SLE patients with high chemokine scores had greater serum IgG reactivity to unmodified and dimethylated (K11Me2) forms of the H2B peptides, compared to patients with low chemokine scores (Fig. 5). While the difference in reactivity to K5Me2 between SLE patients with high and low IFN signatures did not reach statistical significance by ELISA, there was still a strong trend toward greater reactivity in patients who had high IFN signatures. These findings demonstrate that GMR biosensor autoantigen microarrays are capable of identifying autoantibodies associated with dysregulation of the IFN pathway, and defining clinically relevant patient subsets in SLE.

## Discussion

In this study, we developed GMR biosensor autoantigen microarrays and used them to identify autoantibodies associated with chemokine scores and dysregulation of the IFN pathway in SLE. The microarrays were based on GMR biosensor chips, which are capable of measuring local magnetic field changes induced by the presence of MNPs at 72 effective sensors in real time. We validated the microarrays using monoclonal and polyclonal antibodies, and found that the microarrays were capable of sensitive and specific detection of antibodies to recombinant human autoantigens, antigenic peptides, and post-translationally modified peptides corresponding to the N-terminal tail of histone H2B. Real-time measurement of antibody reactivity to post-translational modifications (PTMs) and their consensus binding sequences, as well as point mutants, with GMR biosensor microarrays could allow rapid screening and characterization of hybridomas or cloned antibody libraries.

Previous studies have found associations between IFN gene scores and the presence of specific autoantibodies in SLE^4^,^19^,^20^,^21^,^22^,^23^,^24^,^25^. However, there was variation in the associations between studies, potentially related to differences between patient groups, and the methods used to determine the IFN gene scores and measure antibodies. The autoantibodies associated with chemokine score in SLE are likely more clinically relevant, as chemokine scores were more highly correlated with SLE disease activity, as measured by SLE disease activity index (SLEDAI), systemic lupus activity measure-revised (SLAM-R), erythrocyte sedimentation rate (ESR), and anti-DNA, than the IFN gene score^5^. Further, chemokine score was found to be predictive of disease flare in SLE^6^, while IFN gene score was not^20^. To date, only anti-dsDNA has been associated with chemokine score in SLE^5^,^6^.

To identify autoantibodies associated with chemokine scores and dysregulation of the IFN pathway in SLE, we used GMR biosensor autoantigen microarrays to compare sera from individuals with SLE who had high or low chemokine scores. The microarray analysis revealed that antibody reactivity to 13 autoantigens and peptides was significantly greater in individuals with high chemokine scores, compared to those with low chemokine scores, including histones (H2A and H4, H2B, and H3), methylated and unmodified peptides from the N-terminal tail of H2B, Ribo P, Smith, Ro60, and U1-70K. To our knowledge, these associations were novel and have not been reported previously. We confirmed the association of high chemokine scores with serum reactivity to U1-70K in an independent cohort of individuals with SLE by indirect ELISA. This was in agreement with previous studies that found anti-RNP was associated with increased IFN gene scores^19^,^22^,^23^, and provides more detail as to the specific subunits of the U1-RNP complex that are targeted in the context of dysregulated IFN signaling.

Similar to U1-70K, a number of the other autoantibody associations we observed paralleled previous studies on IFN gene scores in SLE, including dsDNA^4^,^20^,^22^,^23^,^25^, Smith^19^,^20^,^22^, Ribo P^19^, and specific histone proteins (H1, H2A, H2B, and H3/H4)^24^, further confirming their association with dysregulated IFN signaling. Two previous studies found anti-Ro^19^,^22^ was associated with increased IFN gene scores, while another did not^23^. Most clinical tests for Ro antibodies use a combination of two antigens, Ro60 and Ro52, despite the fact that patient sera may have reactivity to either or both of the antigens, and that individual measurement provides clinical information^26^,^27^. Interestingly, we found that SLE patients with high chemokine scores had significantly greater reactivity to Ro60, but not Ro52, compared to patients with low chemokine scores. La antibodies are often observed in combination with anti-Ro in SLE. In agreement with Nikpour *et al*.^23^, and counter to Li *et al*.’s^19^ findings with the IFN gene score, we did not observe a significant association between high chemokine scores and reactivity to La.

The N-terminal tail of H2B is a known autoantigen in SLE^8^,^24^ and PTMs of the tail are involved in regulation of chromatin structure and gene expression^28^,^29^. The GMR biosensor autoantigen microarrays identified novel associations between chemokine scores and serum reactivity to methylated (K5Me1, K5Me2 and K11Me2) and unmodified peptides from the N-terminal tail of H2B in individuals with SLE. ELISA measurements with an independent cohort of SLE patients with high or low IFN signatures demonstrated that serum IgG reactivity to unmodified and dimethylated forms of the H2B N-terminal tail are associated with dysregulation of IFN signaling. Post-translational modifications have been proposed as a mechanism of loss of tolerance to self-antigens^30^,^31^. Further, distinct histone tail modifications are enriched in neutrophil extracellular traps (NETs), a potential self-antigen present at sites of inflammation^32^. Our results suggest that tolerance has been broken to the dimethylated form of the N-terminal tail of H2B. It will be interesting to investigate whether this histone mark is also enriched in NETs.

Limitations of the GMR biosensor autoantigen microarray platform include the number of GMR biosensors on each chip and the technique’s sensitivity. As GMR biosensors are highly scalable (over 100,000 sensors per cm^2^)^15^,^33^, additional sensors will be included on future generations of the chips, increasing the number of autoantigens that can be measured in parallel. Compared to previous use of GMR microarrays to measure soluble proteins using matched capture and detection antibody pairs, the GMR biosensor autoantigen microarrays had lower sensitivity (comparable to ELISA). We are currently investigating immobilization chemistry and blocking as potential factors influencing the technique’s sensitivity.

Advantages of GMR biosensor autoantigen microarrays include that they could be easily adapted to detect autoantibodies in other clinical applications, including additional autoimmune diseases or cancer^34^. Further, GMR biosensors can measure antibody binding in real time, allowing the estimation of the affinity of autoantibodies, and longitudinal studies on autoantibody affinity maturation. Our group is currently developing microfluidic devices for the GMR biosensor microarrays that will enable precise sample handling^35^,^36^, which will enhance precision and streamline affinity studies. Additionally, the microarrays could enable point-of-care (POC) measurement of clinically important antibodies, because the reader station can be easily miniaturized to the size of a smartphone, and measurements can be performed in minutes.

In conclusion, we have developed GMR biosensor autoantigen microarrays and used them to identify novel autoantibody associations with chemokine scores and dysregulation of IFN signaling in SLE. The autoantigens included post-translationally modified histone H2B N-terminal tail peptides. Identifying autoantibodies associated with dysregulation of the IFN pathway in SLE may provide new insights into the mechanisms underlying activation of the IFN pathway and SLE pathogenesis. Further, these findings have the potential to inform ongoing clinical trials of inhibitors of type I IFN signaling in SLE (ClinicalTrials.gov, NCT02446899 & NCT02446912). Future generations of the microarrays have the potential to be real-time clinical proteomics platforms, enabling rapid monitoring of disease activity and prediction of disease flares, allowing preemptive treatment.

## Methods

### Patients

Serum samples from individuals with SLE and normal controls were collected as part of the Autoimmune Biomarkers Collaborative Network (ABCoN). The protocols were approved by the University of Minnesota Institutional Review Board (protocol 0110M09982), and the samples were studied in accordance with the approved guidelines. All individuals with SLE met American College of Rheumatology (ACR) revised criteria for classification of SLE^37^. Written informed consent was obtained from all participants. The chemokine scores of all individuals with SLE had been determined, as previously described^6^.

### GMR biosensor chip

An 8 × 10 array of GMR biosensors was fabricated on a chip with a dimension of 10 × 12 mm (designed by MagArray Inc., CA, USA). Each GMR biosensor consists of multiple stripes of spin valve stack of IrMn/CoFe/Ru/CoFe/Cu/CoFe, and has an area of 100 × 100 μm. The sensors were connected through 300-nm-thick Ta/Au/Ta films in the manner of grid network to electrical pads for external connection with the reader station. A 30-nm-thick passivation layer was deposited on top of the sensors except 8 sensors in the last row, and a 300-nm-thick oxide layer was deposited on the rest of the sensor chip surface to prevent the sensors from corrosion and breakdown. The 8 sensors with the thicker passivation layer were used as electrical reference sensors.

The reader station equipped with a Helmholtz coil generates an AC magnetic field at a frequency of 90 Hz to modulate the magnetization of the sensors. The station also applies an AC current at a frequency of 500 Hz through the sensors to operate them using the double modulation scheme^38^. The details of surface chemistry were described in the previous study^39^.

### GMR biosensor autoantigen microarrays

The autoantigens shown in Supplementary Table 1 were printed at 0.2 mg/mL (in PBS) on the sensors of a GMR sensor chip in replicates of 4 using a non-contact microarrayer (sciFLEXARRAYER, Scienion, NJ, USA). BSA (Sigma-Aldrich) and biotin-BSA (Fisher Scientific) were printed in parallel as quality controls. After printing, the microarrays were incubated overnight in a humid chamber at 4 °C, washed with rinsing buffer (PBS pH 7.4 with 0.1% BSA and 0.05% Tween-20, Sigma-Aldrich), and blocked with 1% BSA for 1 hour. After blocking, the microarrays were washed with rinsing buffer and probed with diluted serum (1/200 in rinsing buffer) or commercial antibodies for 2 hours (at specified concentrations in rinsing buffer) with agitation at room temperature. Commercial antibodies were as follows, anti-FLAG (F1804, Sigma-Aldrich), anti-U1-70K (70R-4901, Fitzgerald Industries International), anti-Ro52 (sc-25351, Santa Cruz Biotechnology), anti-H2B K5Ac (ab61227, Abcam) and anti-H2B K20Ac (ab52988, Abcam). The microarrays were then washed with rinsing buffer and incubated for 1 hour with biotinylated anti-human IgG (109-065-098, Jackson ImmunoResearch, PA, USA; at 100 ng/mL), anti-mouse IgG (ab98711, Abcam, MA, USA; at 50 ng/mL), or anti-rabbit IgG (ab97198, Abcam; 100 ng/mL) secondary antibodies.

For analysis of serum samples with peptides, streptavidin (5 mg/mL in PBS) was printed on the sensors of a GMR sensor chip, along with BSA and purified human IgG (Sigma) controls. After printing, the microarrays were incubated overnight in a humidified chamber at 4 °C, washed with rinsing buffer, blocked with 1% BSA for 1 hour, and washed sequentially with the rinsing buffer and distilled water. The biotinylated peptides shown in Supplementary Table 2 were then printed on the streptavidin-coated sensors using the microarrayer, and the microarrays were incubated overnight in a humid chamber at 4 °C. After washing with rinsing buffer, the microarrays were probed with diluted serum (1/200 in rinsing buffer) for 1 hour with agitation at room temperature.

### Detection of MNPs

Autoantigen microarrays were washed with rinsing buffer and inserted into the reader station. The reader station measured the initial magnetoresistive ratio (MR), which is determined by electrical resistance changes due to an external magnetic field with respect to the nominal resistance, and recorded the baseline signals of each sensor (Supplementary Figure 8). The signals were calculated using a ratio of instantaneous MR to the initial MR. After baseline recording, signals from the sensors were recorded during the addition of 40 μL of streptavidin-coated or Protein G-coated MNPs (130-048-101 and 130-071-101, Miltenyi Biotec, CA, USA). The stray field from bound MNPs changes the magnetic properties of the sensors and induces changes in MR. The signals from BSA- and MCC-coated sensors were considered background of autoantigens and peptides, respectively, and were subtracted from all other sensors’ signals on the microarrays. The electrical read-out system and methodologies were used according to Hall *et al*.^38^.

### ELISA

Nunc-Immuno Maxisorp 96-well plates (Thermo Scientific) were coated with U1-70K at 2 μg/mL in PBS (autoantigen information is shown in Supplementary Table 1). A BSA-coated plate (10 mg/mL) was used to measure non-specific binding. For biotinylated H2B peptides, Streptavidin High Binding Capacity Coated 96-Well Plates (Pierce) were coated with the peptides at 2 μg/mL in PBS. The plates were washed 5 times in wash buffer (PBS with 0.05% Tween-20), before blocking with 10 mg/mL BSA in PBS with 0.05% Tween-20. After washing, patient sera were diluted 1/100 or commercial antibodies were diluted at the indicated concentrations in antibody buffer (10 mg/mL BSA in PBS with 0.05% Tween-20) and incubated overnight at 4 °C. All samples were measured in duplicate wells. After washing, plates were probed with Europium conjugated goat-anti rabbit, rabbit anti-mouse, or mouse anti-human IgG antibodies (Perkin Elmer AD0105, AD0124 and 1244-330) diluted 1/500 in Delfia Assay Buffer (Perkin Elmer). Plates were washed and incubated in Delfia enhancement buffer (Perkin Elmer) for 25 minutes at 37 °C before measuring the time-resolved fluorescence with a Wallac Victor model 1420 Multilabel Counter (Perkin Elmer).

### Statistics

The R programming language (version 3.2.2) was used for analysis^40^. The samr package^41^ was used for SAM analysis with the following settings: nperms = 1000, resp.type = “Two class unpaired”, testStatistic = “wilcoxon” and random.seed = 839938. The pheatmap package was used to render heatmaps^42^. Prism 6 software (GraphPad, La Jolla, CA) was used to perform Mann-Whitney tests (no correction was made for multiple testing).

## Additional Information

**How to cite this article**: Lee, J.-R. *et al*. Multiplex giant magnetoresistive biosensor microarrays identify interferon-associated autoantibodies in systemic lupus erythematosus. *Sci. Rep.* **6**, 27623; doi: 10.1038/srep27623 (2016).

## Supplementary Material

Supplementary Information

## Figures and Tables

**Table 1:** Demographics of individuals with SLE analyzed by GMR biosensor autoantigen microarrays.

Variable	Chemokine high	Chemokine low	p-value
Age at sample, median (range) years	36 (22–62)	42.5 (29–61)	0.2262
Sampling date, post-diagnosis, median (range) years	8 (0–26)	7 (2–23)	0.4433
Number (%) females	15 (100)	15 (100)	1#
ANA titer	960 (160–20480)	320 (40–1280)	0.0025
dsDNA antibody positive patients (%)	15 (100)	3 (21)	<0.0001#
SLEDAI, median (range)	6 (0–16)	0 (0–8)	0.0006
Asian/Pacific Islander (%)	1 (7)	0 (0)	
Non-Hispanic Caucasian (%)	1 (7)	5 (36)	0.1186#
Black (%)	13 (87)	9 (64)	
Chemokine score, median (range)	93 (87–100)	31 (18–40)	<0.0001

Groups were compared by Mann-Whitney test, or Fisher’s exact test (indicated by #).
