# Supplementary material for: Multiplex giant magnetoresistive biosensor microarrays identify interferon-associated autoantibodies in systemic lupus erythematosus
Source: Sci Rep. 2016 Jun 9;6:27623. doi: 10.1038/srep27623 (PMC4899742; doi:10.1038/srep27623)
Supplement: Supplementary Information [file srep27623-s1.pdf]

Supplementary Information

Multiplex giant magnetoresistive biosensor microarrays identify interferon-associated autoantibodies in systemic lupus erythematosus

Jung-Rok Lee, D. James Haddon, Hannah E. Wand, Jordan V. Price, Vivian K. Diep, Drew A. Hall, Michelle Petri, Emily C. Baechler, Imelda M. Balboni, Paul J. Utz, and Shan X. Wang[*,§]

[*] Department of Materials Science and Engineering, Stanford University, Stanford, California, USA
[§]Department of Electrical Engineering, Stanford University, Stanford, California, USA

Address: 476 Lomita Mall, Room 351 McCullough Building, Stanford, CA 94305

Email address: sxwang@stanford.edu

Supplementary Table 1. List of autoantigens and controls used for GMR biosensor microarrays and ELISA.

Name	Catalog number	Vendor
Histone H2A & H4	HIS1002	Immunovision
Histone H2B	HIS1003	Immunovision
Histone H3	HIS1004	Immunovision
Plasmid double-stranded DNA	A12301	Diarect
Ribonucleoprotein 52kDa (Ro52)	A12701	Diarect
Ribonucleoprotein 60kDa (Ro60)	A17401	Diarect
Ribosomal Phosphoprotein P0 (Ribo P)	A14101	Diarect
Sjögren syndrome type B (La/SSB)	A12801	Diarect
Small nuclear ribonuclearprotein 68 (U1-70K)	A13001	Diarect
Smith (Sm)	SMA-3000	Immunovision
Centromere protein A (CENPA)	A16901	Diarect
Biotinylated BSA	PI29130	Fisher Scientific
BSA	P3688	Sigma-Aldrich
Human IgG	I4506	Sigma-Aldrich
Streptavidin	016-000-113	Jackson ImmunoResearch

Supplementary Table 2. List of peptides used for GMR biosensor microarray and ELISA.

Name	Sequence / Catalog number	Modification
FLAG	DYKDDDDK	
FLAG Mut1	DYADDDDK	
FLAG Mut2	DYADDDDA	
FLAG Mut3	AYKADDDK	
FLAG Mut4	AYAADDDK	
H2B 1-7	PEPAKSA	K-biotin
H2B 1-7 K5Ac	PEPAK(Ac)SA	K-biotin
H2B 1-7 K5Me2	PEPAK(Me2)SA	K-biotin
H2B 8-14	PAPKKGS	K-biotin
H2B 8-14 K12Ac	PAPKK(Ac)GS	K-biotin
H2B 8-14 K11Me2	PAPK(Me2)KGS	K-biotin
H2B 1-20	PEPAKSAPAPKKGSKKAVTK	
H2B 1-20 AllAc	PEPAK(Ac)SAPAPKK(Ac)GSK(Ac)KAVTK(Ac)	
H2B 1-20 Mut	PEPAASAPAPAAGSAAAVTA	
H2B 1-21	PEPAKSAPAPKKGSKKAVTKA	N-biotin
H2B 1-21 AllAc	PEPAK(Ac)SAPAPK(Ac)K(Ac)GSK(Ac)K(Ac)AVTK(Ac)A	N-biotin
H2B 1-21 K5Ac	PEPAK(Ac)SAPAPKKGSKKAVTKA	N-biotin
H2B 1-21 K5Me1	PEPAK(Me)SAPAPKKGSKKAVTKA	N-biotin
H2B 1-21 K5Me2	PEPAK(Me2)SAPAPKKGSKKAVTKA	N-biotin
H2B 1-21 K5Me3	PEPAK(Me3)SAPAPKKGSKKAVTKA	N-biotin
H2B 1-21 Mut	PEPAASAPAPAAGSAAAVTAA	N-biotin
H2B 2-21	EPAKSAPAPKKGSKKAVTKA	
H2B 2-21 AllAc	EPAK(Ac)SAPAPKK(Ac)GSK(Ac)KAVTK(Ac)A	
H2B 2-21 Mut	EPAASAPAPAAGSAAAVTAA	
H2B 11-21	KKGSKKAVTKA	
H2B 11-21 AllAc	KK(Ac)GSK(Ac)KAVTK(Ac)A	
H2B 11-21 Mut	AAGSAAAVTAA	
U1-70K 81-100	EVETELKMWDPHNDPNAQGD	
U1-70K 91-110	PHNDPNAQGDAFKTLFVARV	
U1-70K 101-120	AFKTLFVARVNYDTTESKLR	
U1-70K 111-130	NYDTTESKLRREFEVYGPIK	
U1-70K 121-140	REFEVYGPIKRIHMVYSKRS	
Vimentin 256-275	IDVDVSKPDLTAALRDVRQQ	N-biotin
Vimentin 256-275 R273Cit	IDVDVSKPDLTAALRDVR(Cit)QQ	N-biotin
MCC 88-103	ANERADLIAYLKQATK	N-biotin

K(Ac) : acetylated lysine, K(Me): mono-methylated lysine, K(Me2): di-methylated lysine, K(Me3): tri-methylated lysine, R(Cit): citrulline. K-biotin: biotinylated lysine at C-terminus; N-biotin: biotinylated at N-terminus.

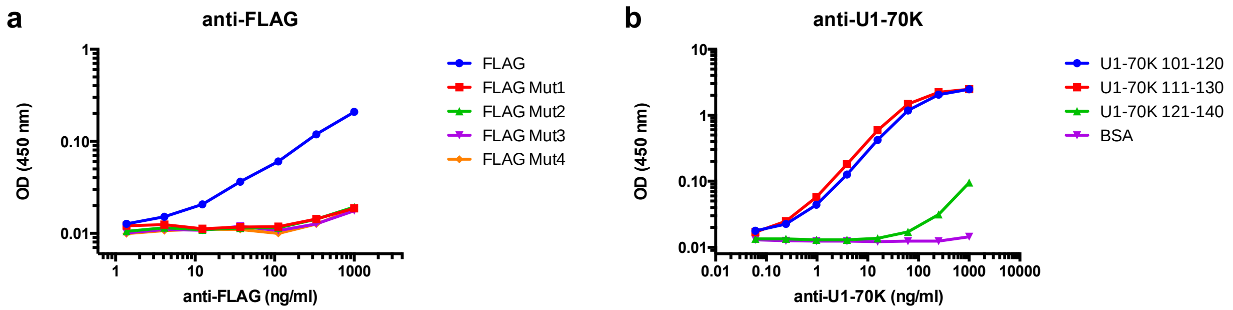

Supplementary Figure 1. Colorimetric ELISA measurement of FLAG and U1-70K antibodies. (a) Anti-FLAG (M2 clone) and (b) Anti-U1-70K antibody (70R-4901, Fitzgerald) were titrated against the FLAG octapeptide or 3 U1-70K peptides, respectively.

.

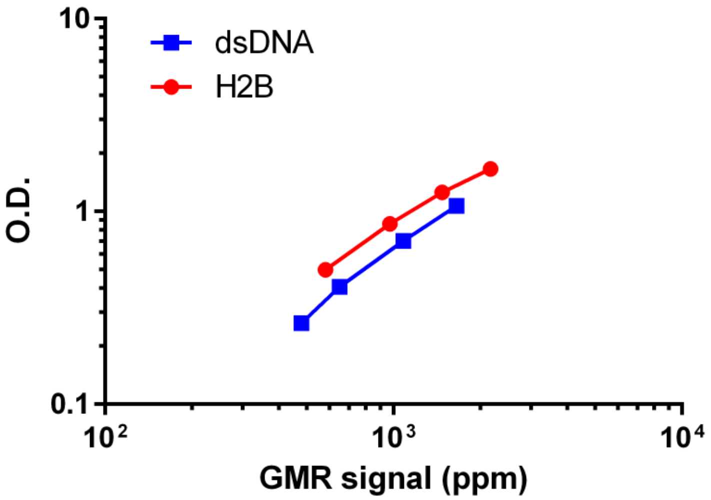

Supplementary Figure 2. Comparison of GMR microarray and ELISA for the detection of serum IgG to charged antigens. Serial dilutions of serum from an SLE patient known to have antibodies to dsDNA and H2B were measured using both GMR biosensors and ELISA. The dsDNA and H2B antigens have negative and positive net charges (at pH 7), respectively. Similar slopes and strong positive correlations were observed between GMR microarray and ELISA measurements for both dsDNA (slope = 6.794×10^{-4} / r = 0.9991) and H2B (slope = 7.304×10^{-4} / r = 0.9946), suggesting that the intrinsic charge of MNPs do not affect detection of charged antigens.

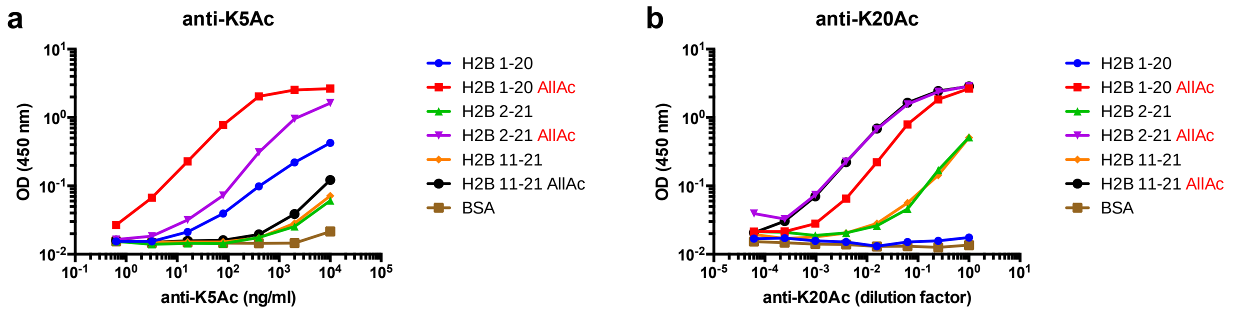

Supplementary Figure 3. Measurement of PTM-specific H2B antibodies by peptide ELISA. Anti-H2B K5Ac (a; ab61227, Abcam) and anti-H2B K20Ac (b; ab52988, Abcam) were measured by peptide ELISA (colorimetric). The concentration of anti-H2B K20Ac antibody was not provided by the manufacturer, and so the relative concentration is shown. Peptides that contain the target epitope are indicated in red.

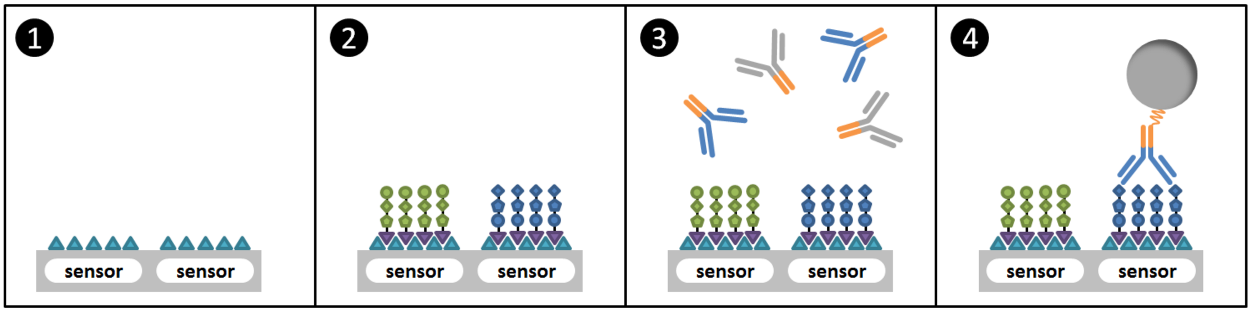

Supplementary Figure 4. Schematic of assaying antibody reactivity to peptides with GMR microarrays, using two-step immobilization. (1) Streptavidin molecules (blue triangles) were printed on each sensor (not to scale). (2) After washing and blocking with BSA, biotinylated peptides were printed on streptavidin-coated sensors. Purple triangles represent biotin. (3) Microarrays were washed and incubated with antibody-containing samples. (4) Following washing, microarrays were inserted into the reader and Protein G-coated MNPs were used to detect antibodies bound to the peptides.

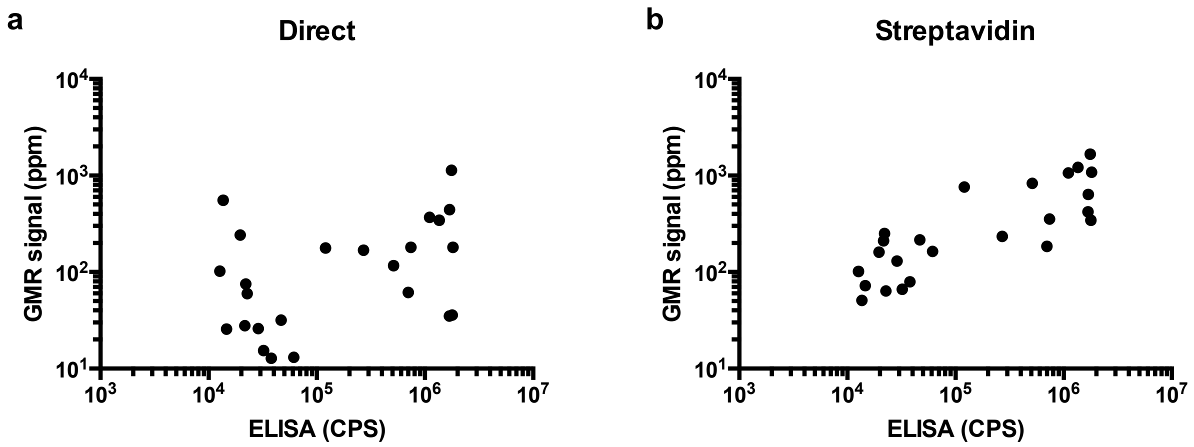

Supplementary Figure 5. Comparison of direct and streptavidin-anchored spotting techniques on GMR biosensor microarrays with peptide ELISA. The IgG reactivity of serum samples from 4 pediatric SLE patients was measured independently with GMR biosensor microarrays with 6 biotinylated peptides either (a) directly spotted on sensors, or (b) spotted on streptavidin-coated sensors. The same sera and peptides (H2B 1-7, H2B 1-7 K5Ac, H2B 1-7 K5Me2, H2B 8-14, H2B 8-14 K12Ac, and H2B 8-14 K11Me2) were used to perform ELISA for comparison.

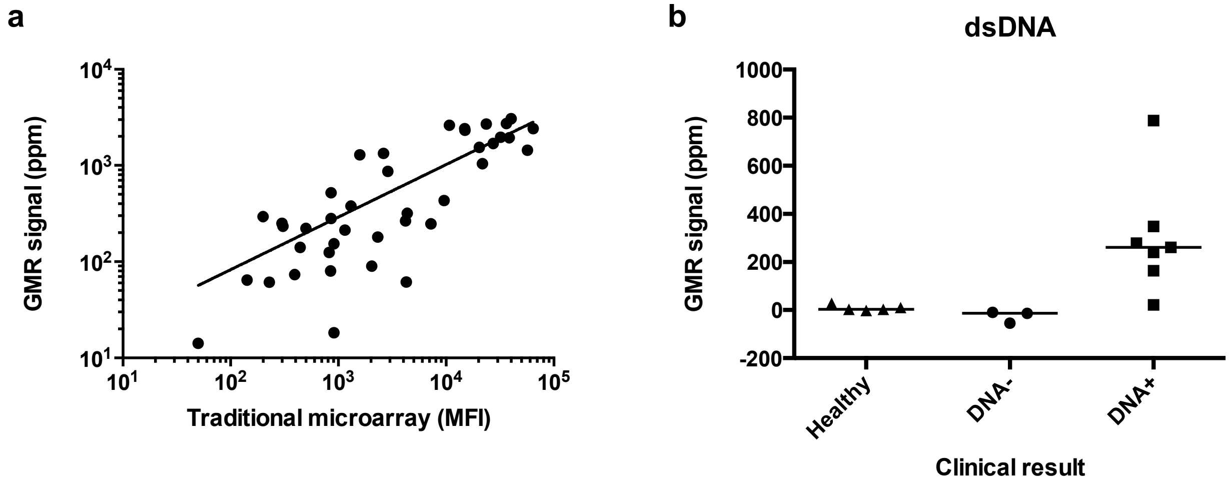

Supplementary Figure 6. Comparison of GMR biosensor autoantigen microarrays with fluorescence-based autoantigen microarrays and clinical antibody tests. (A) Sera from 5 pediatric SLE patients were independently measured with GMR biosensor autoantigen microarrays and fluorescence-based autoantigen microarrays for 8 autoantigens (Histone H2A & H4, Histone 2B, Ro52, Ro60, dsDNA, Ribo P, Smith, and U1-70K), and a high level of agreement was observed ($R^2 = 0.6541$). (B) Sera from 10 pediatric SLE patients and 5 healthy controls were measured using GMR biosensor autoantigen microarrays. The patients were divided based on the results of their clinical anti-dsDNA tests, and GMR measurements of serum IgG reactivity to dsDNA were compared between groups. Serum IgG reactivity to dsDNA was greater in all anti-dsDNA positive patients, compared to negative patients ($p < 0.05$, Mann-Whitney test).

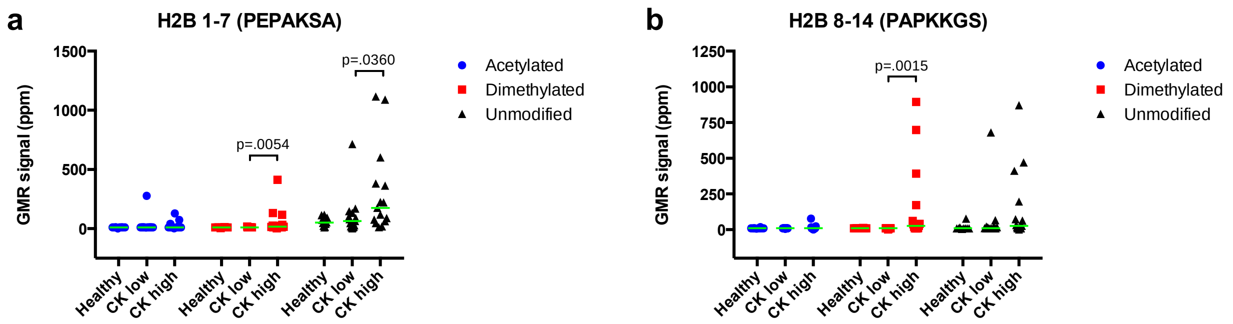

Supplementary Figure 7. GMR biosensor peptide microarray analysis of SLE patients' serum IgG reactivity to peptides derived from the N-terminal tail of histone H2B. Sera from 15 chemokine high and 15 chemokine low SLE patients (and 10 healthy controls) were used to probe GMR biosensor peptide microarrays, and their IgG reactivity to acetylated, dimethylated and unmodified forms of H2B peptides (a, H2B 1-7; and b, H2B 8-14) are shown. Mann-Whitney tests were used to compare groups. Green bars are the average of signals in each group.

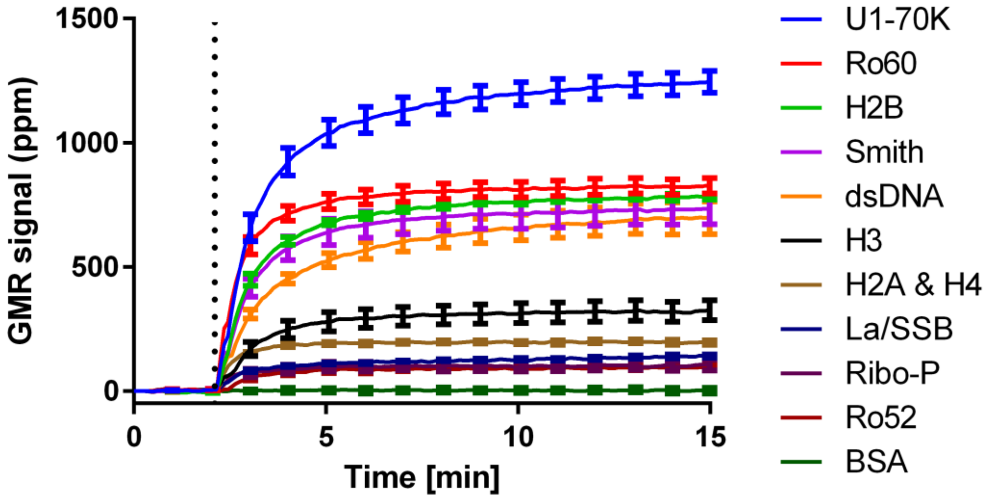

Supplementary Figure 8. Real-time signals of GMR biosensor autoantigen microarrays. A GMR biosensor autoantigen microarray was probed with serum from an individual with SLE, followed by incubation with biotinylated anti-human IgG. The chip was inserted into the reader station, and MNPs were added at ~ 2 min (indicated by the dotted line). Error bars represent standard deviations of signals from 4 identical sensors.

Additional Methods

Colorimetric ELISA (Supplementary Figures 1 and 3): The peptide (either FLAG, FLAG Mut 1 to 4, U1-70K 101-120, 111-130, 121-140, H2B 1-20, H2B 1-20 All Ac, H2B 2-21, H2B 2-21 All Ac, H2B 11-21, and H2B 11-21 All Ac) at 1 µg/mL was added to each well of a 96-well plate, and incubated overnight at 4 °C. The plate was blocked with 1 % BSA for 1 hour, and serial dilutions of commercial antibodies (anti-FLAG, anti-U1-70K, anti-K5Ac, and anti-K20Ac) were added and incubated for 2 hours at room temperature. A washing step with rinsing buffer containing 0.1 % BSA and 0.05 % Tween-20 in PBS was performed between every step. Species-specific detection antibodies (anti-mouse IgG or anti-rabbit IgG) were added to each well, and incubated for 1 hour. Streptavidin-conjugated HRP (Horseradish peroxidase, DY998, R&D Systems) was then added to the plate. After 20 min of incubation, a 1:1 mixture of hydrogen peroxide and tetramethylbenzidine (DY999, R&D Systems) was added to the plate and allowed for color development. A sulfuric acid solution was then added to stop the reaction without washing. The absorbance at 450 nm was measured using a microplate reader (Infinite 200 Pro, Tecan).

Fluorescent microarray (Supplementary Figure 5a): Autoantigens were printed on the microarrays, and incubated overnight. The microarrays were washed three times in PBST (PBS with 0.05 % Tween-20) and then blocked with PBST + 3% fetal calf serum (FCS) for 30 min at room temperature. After washing in PBST, the microarrays were loaded with serum samples and incubated overnight at 4 °C. The microarrays were then washed three times in PBST, and human IgG-specific Cy5-conjugated antibody (109-495-129, Jackson ImmunoResearch) was applied to the microarrays and incubated for 45 min at room temperature. The microarrays were then washed with PBST and distilled water sequentially before dried in a rack centrifuged at 300 g for 5 min. The microarrays were immediately scanned using an Axon digital scanning system and Genepix Pro 6.1 software (Molecular Devices).
